# Distinct Intraspecies Variation of *Cutibacterium acnes* and *Staphylococcus epidermidis* in Acne Vulgaris and Healthy Skin

**DOI:** 10.3390/microorganisms13020299

**Published:** 2025-01-29

**Authors:** Tina Hamann, Holger Brüggemann, Cecilie Feidenhansl, Erinda Rruci, Julia Gallinger, Stefan Gallinat, Jennifer Hüpeden

**Affiliations:** 1 Beiersdorf AG Research & Development, Discovery, 20245 Hamburg, Germany; julia.gallinger@beiersdorf.com (J.G.); stefan.gallinat@beiersdorf.com (S.G.); jennifer.huepeden@beiersdorf.com (J.H.); 2 Department of Biomedicine, Aarhus University, 8000 Aarhus, Denmark; brueggemann@biomed.au.dk (H.B.); cecilief@biomed.au.dk (C.F.); erindarruci@biomed.au.dk (E.R.)

**Keywords:** skin microbiome, *Cutibacterium acnes*, acne vulgaris, *Staphylococcus*, *Staphylococcus epidermidis*, amplicon-based next-generation sequencing

## Abstract

Human skin hosts a diverse array of microorganisms that contribute to its health. Key players in the facial skin microbiome include *Cutibacterium acnes* and staphylococci, whose colonization patterns may influence dermatological conditions like acne vulgaris. This study examined the facial microbiome composition of 29 individuals, including 14 with moderate to severe acne and 15 with healthy skin, using single locus sequence typing (SLST) amplicon sequencing. The results showed a shift in the relative abundances of *C. acnes* phylotypes: SLST types A, C, and F were increased in acne, while types H, K, and L were reduced compared to healthy skin. Among staphylococci, the relative abundance of *S. epidermidis*, *S. capitis*, and *S. saphrophyticus* increased in acne, while *S. saccharolyticus* and *S. hominis* decreased. The amplicon sequencing approach could also identify a population shift of *S. epidermidis*: a specific *S. epidermidis* phylogenetic lineage (type 3) was reduced in acne, while two abundant lineages (types 1 and 2) were elevated. These findings suggest that distinct phylogenetic lineages of both *C. acnes* and *S. epidermidis* are linked to healthy versus diseased skin, highlighting a potential role for both microorganisms in disease prevention and aggravation, respectively.

## 1. Introduction

Acne vulgaris remains one of the most prevalent skin conditions worldwide, impacting the quality of life of millions of people. Multiple factors contribute to acne, including increased sebum production, follicular hyperkeratinization, dysbiosis of the skin microbiome, and an inflammatory cascade [1]. *Cutibacterium acnes* (*C. acnes*), a Gram-positive bacterium that typically resides within sebaceous follicles of human skin, is regarded as an important player in acne [2,3,4,5,6,7,8,9,10,11,12]. However, *C. acnes* is also a ubiquitous colonizer of healthy skin [2,3,4]. In recent years it could be determined that different *C. acnes* phylotypes are enriched on healthy and acneic skin, respectively [9,10,11,12,13,14,15]. This has been confirmed in culture-dependent as well as culture-independent studies. For culture-independent studies, different techniques were applied, including amplicon-based next-generation sequencing (NGS) [14] and shotgun metagenome sequencing [15]. The use of amplicon-based NGS to determine the relative abundance of *C. acnes* phylotypes in a given skin sample such as a skin swab takes advantage of the well-resolved population structure of *C. acnes.* The *C. acnes* population can be divided into different subspecies and phylotypes, namely IA1, IA2, IB, IC, II, and III [12,13]. Mixed populations of *C. acnes* can be analyzed with a single locus sequence typing (SLST) scheme that enables the differentiation into ten classes (A to L) [16]. SLST classes A to E correspond to phylotype IA1 strains, whereas SLST classes F, G, H, K, and L correspond to phylotypes IA2, IC, IB, II, and III, respectively. Some SLST classes are associated with acne, whereas others have been identified as markers of healthy skin [11,12,13,14,17]. Acne-associated phylotypes include SLST classes A and C (both phylotype IA1) and F (IA2), whereas healthy skin is colonized with more diverse populations with higher relative abundances of strains belonging to the SLST classes H (IB) and K (II). Acne- and healthy skin-associated phylotypes of *C. acnes* can differ in their inflammatory potential and their interaction with immune cells [18,19,20]. The underlying mechanisms are incompletely understood. Current data suggest that different phylotypes can express and produce different (levels of) inflammatory proteins or molecules, such as porphyrins, adhesins, CAMP factors, lipases, hyaluronidases, and others. The reader is referred to recent reviews for more details [8,11,12,13,21]. Besides *C. acnes*, staphylococci are the second most abundant group of bacteria on skin surfaces of the upper body. *Staphylococcus epidermidis* (*S. epidermidis*), a coagulase-negative species, is particularly noteworthy for its abundance and diverse roles as a commensal organism. It can be part of the skin’s natural defense mechanism, creating a protective barrier against the colonization of potentially harmful pathogens [22,23]. The species is phylogenetically divided into main clades and is assigned to different sequence types (ST) [24,25,26,27], which refers to a classification based on the sequences of a number of *S. epidermidis* housekeeping genes, to identify genetic relationships and track strains [28]. Notably, some *S. epidermidis* STs such as ST2, ST5, ST23, and ST215 have been linked to nosocomial infections, suggesting, at least to some extent, ST-specific differences in the pathogenic potential of *S. epidermidis* [27,29,30,31]. The lack of an SLST-based scheme to resolve the population of *S. epidermidis* in skin samples is limiting efforts to identify and study a potential *S. epidermidis* population dysbiosis in skin diseases such as acne. However, it was previously found that the *tuf* gene, present in all staphylococci and encoding the Tu elongation factor, is a useful phylogenetic marker to differentiate staphylococcal species [32,33,34,35]. We have further identified a fragment of the *tuf* gene, designed *tuf2*, and showed that the *tuf2* amplicon NGS allows the unambiguous identification of staphylococcal species and the distinction of phylogenetic clades of *S. epidermidis* [36]. The potential use of the *tuf2* scheme to distinguish phylogenetic lineages of *S. epidermidis* has not been investigated so far in skin swab samples.

Understanding the differences in the colonization patterns of staphylococci, alongside *C. acnes*, in acne-prone and healthy skin is essential for deciphering the multifaceted interactions within the skin microbiome. This study aimed to determine differences in the community structures of *C. acnes* and staphylococci in individuals with moderate/severe acne compared to those with healthy skin.

## 2. Materials and Methods

### 2.1. Cohort and Skin Sampling

Swab samples were collected from 29 female volunteers (acne-prone, *n* = 14; healthy, *n* = 15) with an age range of 20–44 years from two test sites on the forehead, as described previously [14] (Supplementary Table S1). The acne severity of the individuals was graded according to the Clinical Expert Grading Scale (EXG), developed internally at the institute. EXG scores from 0 to 6 exist; their meaning is further specified in Supplementary Table S2. All included acne-affected participants had an EXG score ≥ 4 (Supplementary Table S2). In brief, an area of 9 cm^2^ of the forehead was swabbed with a cotton swab, which was pre-moistened in aqueous sampling buffer (50 mM Tris-HCl, 1 mM EDTA, pH 8.0, and 0.5% Tween-20). The swab was vigorously shaken in a tube containing 2 mL of sampling buffer and then removed. The sample was stored at −80 °C before DNA extraction. None of the volunteers had undergone treatment with topical medicine or antibiotics in the last six months. Written informed consent was obtained from all volunteers, and the study was approved by the Institutional Ethics Committee, Bucharest, Romania (10/2020; Study no. 70037).

### 2.2. DNA Extraction, Polymerase Chain Reaction (PCR), and Sequencing

Prior to DNA extraction, skin swab samples were centrifuged (8000× *g*, 30 min at 4 °C), and the supernatant was discarded. The pellets were lysed by using lysostaphin (0.05 mg/mL, Sigma, Burlington, VT, USA) and lysozyme (9.5 mg/mL, Sigma, Burlington, VT, USA) for 1 h. DNA was extracted using the DNeasy PowerSoil Kit (Qiagen, Hilden, Germany), following the manufacturer’s instructions. DNA concentrations were measured with the Qubit dsDNA HS Assay (ThermoFisher Scientific, Waltham, MA, USA) using a Qubit fluorometer. The *tuf2* PCR (for staphylococcal population analysis) was performed as described previously [14] using the primers *tuf2*_fw, 5′-ACAGGCCGTGTTGAACGTG-3′ and *tuf2*_rev, 5′-ACAGTACGTCCACCTTCACG-3′. The SLST amplicon fragment (for *C. acnes* population analysis) was amplified using the primers 5′-TTGCTCGCAACTGCAAGCA-3′ and 5′-CCGGCTGGCAAATGAGGCAT-3′. PCR reaction mixtures were made in a total volume of 25 µL and comprised 5 µL of DNA sample, 2.5 µL AccuPrime PCR Buffer II (Invitrogen, Waltham, MA, USA), 1.5 µL of each primer (10 µM) (DNA Technology, Risskov, Denmark), 0.15 µL AccuPrime Taq DNA Polymerase High Fidelity (Invitrogen, Waltham, USA), and 14.35 µL of PCR grade water. The PCR reaction was performed using the following cycle conditions: initial denaturation at 94 °C for 2 min, 35 cycles of denaturation at 94 °C for 20 sec, annealing at 55 °C for 30 sec, elongation at 68 °C for 1 min, and final elongation step at 72 °C for 5 min. PCR products were verified on an agarose gel and purified using the Qiagen GeneRead^TM^ Size Selection kit (Qiagen, Hilden, Germany). The concentration of the purified PCR products was measured with a NanoDrop 2000 spectrophotometer (ThermoFisher Scientific, Waltham, MA, USA).

Specific indices and Illumina adapters were attached to the amplicons using the Nextera XT Index kit (Illumina, San Diego, CA, USA). Index PCR was performed using 5 µL of template PCR product, 2.5 µL of each index primer, 12.5 µL of 2× KAPA HiFi HotStart ReadyMix, and 2.5 µL PCR grade water. The thermal cycling scheme was as follows: 95 °C for 3 min, 8 cycles of 30 s at 95 °C, 30 s at 55 °C, and 30 s at 72 °C and a final extension at 72 °C for 5 min. Quantification of the products was performed using the Quant-iT dsDNA HS assay kit and a Qubit fluorometer (Invitrogen GmbH, Karlsruhe, Germany) following the manufacturer’s instructions. MagSi-NGSPREP Plus Magnetic beads (Steinbrenner Laborsysteme GmbH, Wiesenbach, Germany) were used for purification of the indexed products as recommended by the manufacturer, and normalization was performed using the Janus Automated Workstation from Perkin Elmer (Perkin Elmer, Waltham, MA, USA). Sequencing was conducted with an Illumina MiSeq platform using dual indexing and the MiSeq reagent kit v3 (600 cycles) as recommended by the manufacturer.

### 2.3. Bioinformatics

Sequences (FASTQ format) obtained after demultiplexing the reads and trimming the primers (Cutadapt v. 3.7; [37]) were processed with QIIME2 (v.2023.5) [38]. Paired-end reads were denoised with DADA2 via q2-dada2 [39]; reads with more than two expected errors in either the forward or reverse reads were discarded, and chimeras were removed. Unique sequences obtained through DADA2 were clustered with VSEARCH [40] using q2-vsearch [41] at a 99% identity cut-off against allele databases. All paired-end reads that passed the quality filters were searched against allele databases at a 99% identity cut-off. The database for the staphylococcal amplicon scheme contained all *tuf2* alleles from staphylococcal genomes available in GenBank (as of December 2023). The allele database for the *C. acnes* SLST amplicon scheme contained over 200 alleles; it is available online (http://medbac.dk/slst_server_script.html, accessed on 15 October 2024). Data were normalized, and low abundant features were filtered. Visualization and statistical analyses were conducted in R (v. 4.3.0) using the packages ggplot2 (v. 3.4.2) [42], phyloseq (v 1.44.0) [43], vegan (v. 2.6–4) [44], and ggpubr (v. 0.6.0).

Phylogenetic analysis was performed with MEGA (v.11) [45]. The *tuf2* alleles were aligned with MUSCLE [46] using default parameters and phylogeny was reconstructed using the Neighbor-Joining method (test of phylogeny: Bootstrap method with 500 replications) [47]. Parsnp (v.1.7.4) was used for whole genome phylogenetic reconstruction [48]. Default settings were used. Phylogenetic trees were visualized using the Interactive Tree Of Life [49]. The 69 *S. epidermidis* genomes used for phylogenomic analysis and reconstruction of the *tuf2* phylogeny were taken from Ahle et al. [17].

### 2.4. Statistical Testing

Statistical testing and figures presenting the amplicon data were generated in GraphPad (prism v10). The Wilcoxon rank-sum test was used for all comparisons between healthy and acne cohorts, with statistical thresholds of 0.001 (***), 0.01 (**), and 0.05 (*).

## 3. Results

### 3.1. Clinical Examination of the Experimental Area and Global Assessment of the Skin State

Before sampling, a clinical examination of the face (except the nasal pyramid, the vermilion border, the crease in the chin, and the rim of the scalp) of participants with acne-prone skin (*n* = 14) was performed to count retentional (R) elements (non-inflammatory comedones and microcysts) and inflammatory (I) acne elements (papules and pustules), resulting in a global assessment of the skin state according to the EXG scale (Supplementary Tables S1 and S2). All test subjects included in the control skin group (*n* = 15) had healthy and unblemished skin: R = 0, I = 0, EXG score = 0. Samples for amplicon-based NGS analysis were taken from all 29 volunteers (acne-prone, *n* = 14; healthy, *n* = 15) on two sites of the forehead.

### 3.2. Determination of the C. acnes Phylotype Dysbiosis

**Figure 1:**
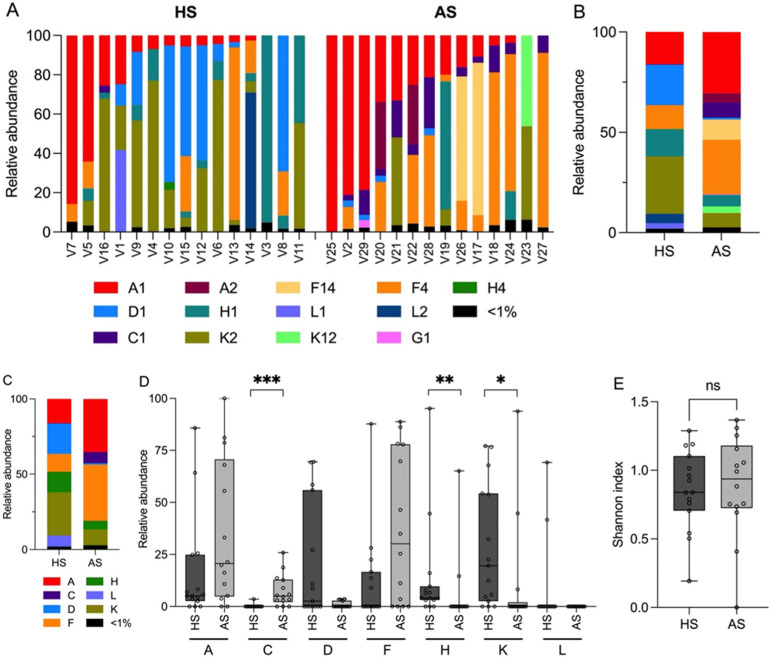
*C. acnes* phylotype composition in the healthy and acne cohort. (**A**) SLST amplicon sequencing results of 15 and 14 skin swab samples from healthy individuals and acne patients, respectively. (**B**) Mean relative abundance of the different *C. acnes* SLST types for the two cohorts. HS, healthy skin; AS, acneic skin. (**C**) Mean relative abundance of the different *C. acnes* SLST classes for the two cohorts. (**D**) Boxplots for the seven most prevalent SLST classes, showing the variation across samples. The midline of the boxplot represents the median, the upper line represents the upper quartile, and the lower line represents the lower quartile. Wilcoxon rank-sum test; ***: *p*-value < 0.001; **: *p*-value < 0.01; *: *p*-value < 0.05; ns, non-significant (*p*-values: A, 0.0846; C, 0.0004; D, 0.2242; F, 0.0724; H, 0.0076; K, 0.0209; L, 0.2241). (**E**) Shannon diversity index (alpha diversity) for the *C. acnes* population in the acne cohort compared to the healthy cohort. Wilcoxon rank-sum test (*p*-value 0.78).

All 29 DNA samples of the collected swabs were analyzed by amplicon-based NGS using the *C. acnes* SLST scheme (Figure 1A). Thirteen SLST types belonging to eight SLST classes were detected; their average relative abundances differed between the healthy skin (HS) and the acneic skin (AS) groups (Figure 1B,C). The mean relative abundances of a few SLST types changed strongly between HS and AS: A1, 16.1% (HS) vs. 30.6% (AS); F4, 11.8% (HS) vs. 27.2% (AS); K2, 28.6% (HS) vs. 7.2% (AS); H1, 13.4% (HS) vs. 5.7% (AS); D1, 20.1% (HS) vs. 0.9% (AS); F14, 0.0% (HS) vs. 10.1% (AS); C1, and 0.2% (HS) vs. 7.5% (AS). The increase in SLST type F14 in AS is due to their abundance in two AS samples. Overall, the data showed an increased relative abundance of SLST classes A, C, and F in AS compared to HS and a decrease in SLST classes D, H, K, and L. The increased relative abundance of SLST class C and the decrease in SLST classes H and K were statistically significant (Figure 1D). The alpha diversity (Shannon index) did not change significantly between AS and HS (Figure 1E).

### 3.3. Determination of the Staphylococcal Populations on Acneic and Healthy Skin

**Figure 2:**
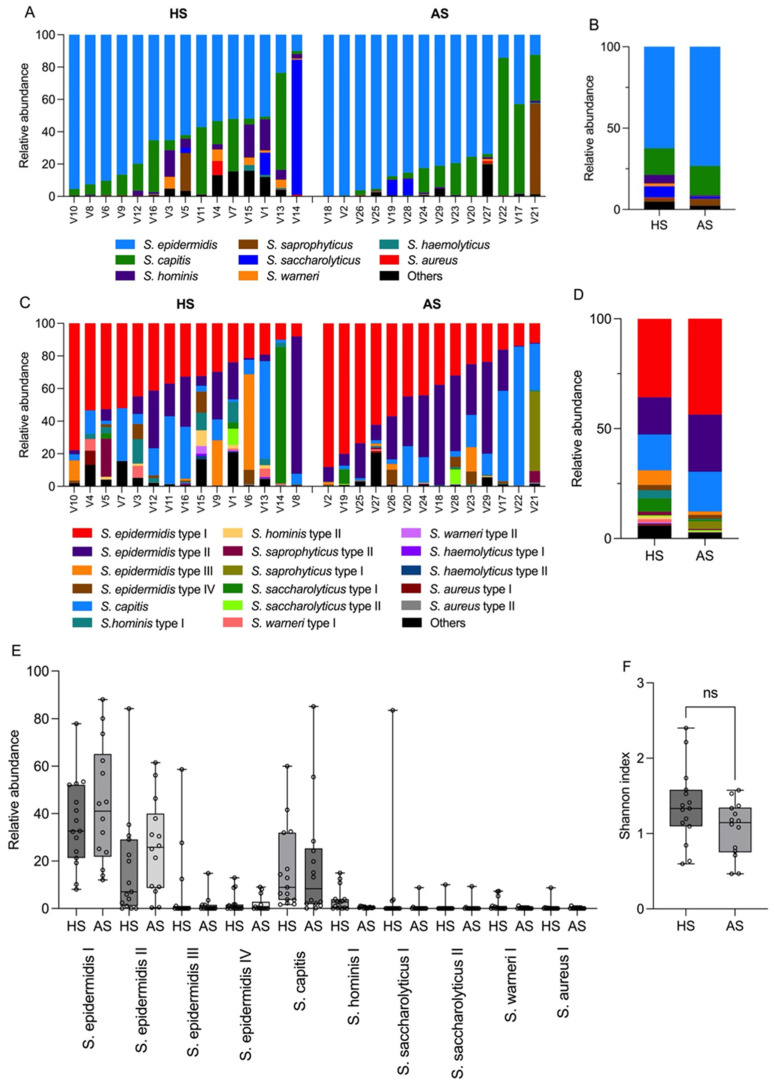
Staphylococcal community composition in the healthy and acne cohort. (**A**) *tuf2* amplicon sequencing results of 15 and 14 skin swab samples from healthy individuals and acne patients, respectively. (**B**) Mean relative abundances of the main staphylococcal species in the two cohorts. HS, healthy skin; AS, acneic skin. (**C**) The composition of the staphylococcal population according to the main identified *tuf2* alleles. Some species are represented by more than one *tuf2* allele, e.g., *S. epidermidis* with four *tuf2* alleles. (**D**) Mean relative abundance of the staphylococcal population at the subtype level of the two cohorts. (**E**) Boxplots for the 10 most prevalent staphylococcal *tuf2* alleles, showing the variation across samples. The midline of the boxplot represents the median, the upper line represents the upper quartile, and the lower line represents the lower quartile. The Wilcoxon rank-sum test showed no significant difference between HS and AS. (**F**) Shannon diversity index (alpha diversity) for the staphylococcal population in the acne cohort compared to the healthy cohort. Wilcoxon rank-sum (*p*-value, 0.1023).

Next, the *tuf2* amplicon scheme was used to determine the relative abundances of staphylococcal species. *S. epidermidis* was the most abundant species on HS (61.9%) and AS (72.9%) among the staphylococci, followed by *S. capitis* (HS, 16.2%; AS, 18.1%), *S. saccharolyticus* (HS, 6.7%; AS, 1.3%), *S. hominis* (HS, 5.2%; AS, 0.4%), and *S. saphrophyticus* (HS, 1.5%; AS, 4.0%) (Figure 2A,B). Whereas the relative abundance of *S. epidermidis*, *S. capitis*, and *S. saphrophyticus* was increased on AS, a decrease was seen for *S. saccharolyticus* and *S. hominis*. The mean relative abundance of *S. aureus* was below 1% in the healthy and acne cohorts.

**Figure 3:**
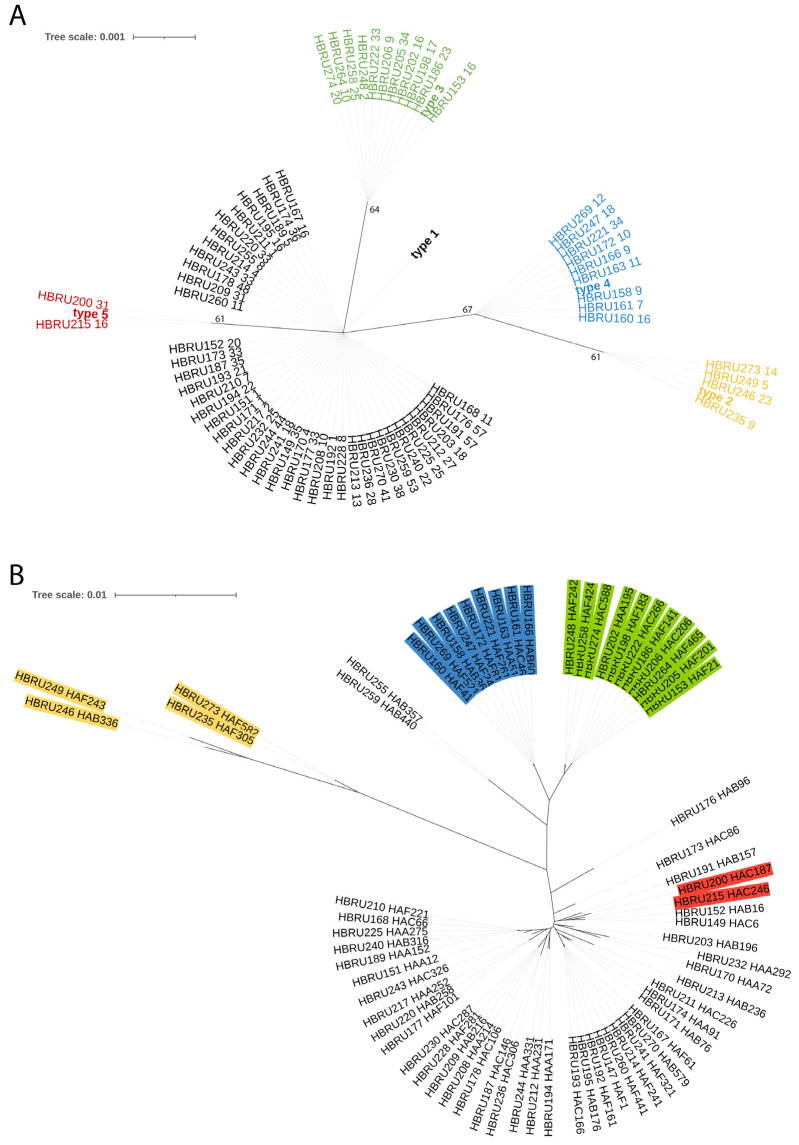
The staphylococcal *tuf2* gene fragment can serve as a phylogenetic marker of *S. epidermidis*. (**A**) The *tuf2* sequences were extracted from 69 *S. epidermidis* genomes (taken from [17]) and phylogenetically compared. Five *tuf2* alleles were found among the 69 strains (alleles (=types) 2–5 are color-coded (type 2, yellow; type 3, green; type 4, blue; type 5, red; type 1 is not color-coded). Bootstrap support (in %) is added to the branches. (**B**) Core genome phylogeny was reconstructed from the 69 *S. epidermidis* strains. Strains were color-coded according to their *tuf2* alleles (color-code see (**A**)). Some phylogenetically distinct lineages are represented by different *tuf2* alleles, e.g., types 2, 3, and 4. Types 1 and 5 are intermixed.

For some staphylococcal species, more than one *tuf2* allele belonged to the same species. Regarding *S. epidermidis*, a search in 69 genomes of *S. epidermidis* taken from the study of Ahle et al. [17] revealed five different *tuf2* alleles (Figure 3A). To investigate if these five *tuf2* alleles were found in distinct phylogenetic lineages of *S. epidermidis*, a core genome phylogenetic reconstruction was performed with the 69 genomes. It was found that the *tuf2* gene fragment could serve as a phylogenetic marker to differentiate four phylogenetic lineages of *S. epidermidis*, subsequently named types 1 to 4 (Figure 3B). Amplicon sequencing could identify four different *tuf2* alleles belonging to *S. epidermidis*, each representing one of the four types of *S. epidermidis* (Figure 2C). Type 1 is the most abundant *S. epidermidis* type (HS, 35.8%; AS, 43.6%), followed by type 2 (HS, 16.9%; AS, 25.9%) and type 3 (HS, 6.6%; AS, 1.5%) (Figure 2D). The two more abundant types (1 and 2) were increased in AS, whereas the low abundant type 3 (and type 4) was reduced in AS (Figure 2E). Although these tendencies were found, they were not statistically significant. The Shannon index was increased on HS versus AS but it is not statistically significant (Figure 2F).

## 4. Discussion

The study’s findings highlight a microbial imbalance in acne-prone skin compared to healthy skin. Specifically, *C. acnes* populations showed notable shifts, with SLST classes A, C, and F being more abundant in acne, while classes D, H, and K were reduced. Statistically significant changes included an increase in SLST class C and a decrease in classes H and K. These results align with previous studies, which reported a relative increase in IA1 strains (SLST classes A–E) and a decline in IB (SLST H) and II (SLST K) strains in acne, despite differences in the typing methods used [9,10,11,12,13,14,15,50,51,52,53]. What are the functional implications of such a *C. acnes* phylotype shift for skin health? It has been found that acne-associated strains of the IA1 clade seem to have a higher inflammatory potential than other *C. acnes* strains [18,19,20,53]. The reason is not fully understood. The (increased) production of proinflammatory molecules such as porphyrins or extracellular vesicles could be one reason [18,19,20]. Moreover, strains of SLST class C that contained a linear plasmid were found to be enriched in acne lesions; these strains had a higher inflammatory potential compared to plasmid-negative strains, indicating that the plasmid could encode additional traits with proinflammatory consequences [53]. One other interesting difference between type IA strains (including the acne-associated SLST classes A, C, and F) and type IB/II strains (comprising the healthy skin-associated SLST classes H and K) concerns the hyaluronic acid lyase (HYL), which is involved in the degradation of hyaluronic acid (HA), a major component of the extracellular matrix in the dermis and epidermis. *C. acnes* expresses different HYL variants: a highly active HYL-IB/II variant produced by strains of phylotypes IB and II that leads to complete degradation of HA and a less active variant HYL-IA produced by strains of phylotype IA, which only partially degrades HA [54]. A recent study found that HA fragments produced by the HYL-IA variant induce a strong TLR2-dependent inflammatory response, in contrast to HA products by HYL-IB/II activity, which leads to a reduced inflammatory response [55]. There are other possible reasons why type IB/II strains seem to be health-beneficial. For example, type IB strains can produce cutimycin, a bacteriocin that is able to control staphylococcal populations in hair follicles [56]. In terms of staphylococcal populations, *S. epidermidis, S. capitis*, and *S. saphrophyticus* were more prevalent on acne-prone skin, while *S. saccharolyticus* and *S. hominis* were reduced, consistent with earlier findings [14]. It has previously been suggested that specific strains of *S. hominis* can be health-beneficial by fostering colonization resistance; some strains can produce specific bacteriocins [57] and some strains can produce autoinducing peptides that can inhibit the *agr* quorum sensing machinery of *S. aureus* [58]. While the functional implications of *S. saccharolyticus* skin colonization are unknown, it is noteworthy that some strains harbor a 55 kb plasmid that encodes an antimicrobial gene cluster identical to the recently identified epifadin gene cluster found in some strains of *S. epidermidis* [59], indicating that some strains of *S. saccharolyticus* are also involved in fortifying the skin barrier through enhancing colonization resistance.

The role of *S. epidermidis* in acne is unknown, but a few publications have indicated that *S. epidermidis* may be a contributing factor [60,61]. One study involved human skin explants co-cultured with bacterial suspensions containing varying ratios of *S. epidermidis* and *C. acnes* [61]. It was found that cultures dominated by *S. epidermidis* induced higher levels of IL-6 compared to those where *C. acnes* predominated. This suggests that an increased relative abundance of *S. epidermidis* and/or the expansion of certain *S. epidermidis* strains could be linked to a heightened inflammatory potential of the skin microbiome. Previous research has not examined potential differences in *S. epidermidis* strains on AS compared to HS. It is well known that *S. epidermidis* is a heterogeneous species; strains can have very different functionalities, including those that maintain skin homeostasis and others that act as opportunistic pathogens [23,24,25,26,27]. Thus, examining *S. epidermidis* to strain-level resolution is important to determine a potential dysbiosis in acne and for subsequent functional studies. Shotgun metagenomics would be a method that could unravel dysbiosis, but this has not been described for *S. epidermidis* so far to our knowledge. An SLST approach like the existing one for *C. acnes* has not been developed for *S. epidermidis*; a two-amplicon approach is available [30], which cannot be easily used for complex samples like skin swabs. Here, we described the use of the *tuf2* gene fragment as a possible approach to distinguish at least the main types of *S. epidermidis* in complex samples. Four distinct *S. epidermidis* lineages—represented by specific *tuf2* alleles—were found in skin samples. *S. epidermidis* type 1 was the most abundant type on healthy and acneic skin. This type and also type 2 were further increased in acne. Interestingly, *S. epidermidis* type 3 decreased in acne. This could indicate that different *S. epidermidis* lineages play distinct host-beneficial or -detrimental roles. Future research is needed to explore the genotypic and phenotypic characteristics of healthy skin-associated type 3 strains and acne-associated types 1 and 2. Interactions between specific lineages/strains of *S. epidermidis* and *C. acnes* likely exist; previous research has found *S. epidermidis* strains with antimicrobial activities that co-colonize healthy skin with *C. acnes* SLST types H and K [17]. Thus, specific strain/type combinations of *S. epidermidis* and *C. acnes* could pave the way for skin microbiome homeostasis. Understanding these microbial interdependencies and interferences could inform targeted treatments for improving skin health.

## 5. Conclusions

The study reveals shifts in the *C. acnes* and *S. epidermidis* populations of acne-prone skin compared to healthy skin. The findings suggest that certain strains of *C. acnes* but also *S. epidermidis* contribute to acne pathologies, possibly via acceleration of inflammation, while other phylogenetically distinct strains of *C. acnes* and *S. epidermidis* have protective roles, possibly by controlling microbial skin colonization via colonization resistance and other so far unexplored mechanisms. Future research focusing on strain-level interactions and their functional roles could provide insights for developing targeted and personalized treatments to restore and/or maintain skin health.

## Data Availability

The amplicon-based NGS data are stored at SRA with the bioproject number PRJNA1173390 and can be accessed at https://www.ncbi.nlm.nih.gov/bioproject/PRJNA1173390 (accessed on 16 October 2024).

## Supplementary Materials

The following supporting information can be downloaded at https://www.mdpi.com/article/10.3390/microorganisms13020299/s1: Table S1: Features of the cohorts used in this study; Table S2: Overview of the distribution of inflammatory and non-inflammatory lesions based on clinical examination in the acne-prone skin group.
