# Supplementary material for: Distinct Intraspecies Variation of Cutibacterium acnes and Staphylococcus epidermidis in Acne Vulgaris and Healthy Skin
Source: Microorganisms. 2025 Jan 29;13(2):299. doi: 10.3390/microorganisms13020299 (PMC11858094; doi:10.3390/microorganisms13020299)
Supplement: Supplementary file 1 [file microorganisms-13-00299-s001.zip › microorganisms-3393252-supplementary.pdf]

Supplementary Table S1: Features of the cohorts used in this study

Age (*years old*)	Test subjects		
	Whole panel	Group A (*acne score ≥ 4)*	Group B (*acne score 0)*
Minimum	20	20	26
Maximum	44	36	44
Mean	33	27	38
Median	32	29	38

Criteria	Test subjects					
	Whole panel		Group A (*acne score ≥ 4*)		Group B (*acne score = 0*)	
	N°	%	N°	%	N°	%
**Phototype**						
II	10	34.5	4	28.6	6	40.0
III	13	44.8	8	57.1	5	33.3
IV	6	20.7	2	14.3	4	26.7
**Sex**						
Female	29	100.0	14	100.0	15	100.0
**Types of skin on face**						
Oily	4	13.8	4	28.6	0	0
Combination skin with oily tendency	8	27.6	5	35.7	3	20.0
Normal skin	10	34.5	4	28.6	6	40.0
Dry skin	2	6.9	0	0	2	13.3
Combination skin with dry tendency	5	17.2	1	7.1	4	26.7
**Body Mass Index (BMI)**						
BMI < 40	29	100.0	14	100.0	15	100.0
**Healthy skin on the test sites**						
Healthy skin on the test sites	29	100.0	14	100.0	15	100.0

Supplementary Table S2: Overview of the distribution of inflammatory and non-inflammatory lesions based on clinical examination in the acne-prone skin group

No	Acne lesion		Number of lesions				Total per type	Total acne lesions	EXG score *
			forehead	left cheek	right cheek	chin			
2	R [1]	Comedones	4	4	4	4	16	38	4
		Microcysts	4	6	3	3	16		
	I [2]	Papules	0	1	0	2	3		
		Pustules	0	1	1	1	3		
17	R	Comedones	3	10	13	10	36	73	4
		Microcysts	6	7	4	4	21		
	I	Papules	0	5	6	1	12		
		Pustules	1	1	2	0	4		

18	R	Comedones	8	15	15	11	49	93	6
		Microcysts	10	4	5	4	23		
	I	Papules	0	5	4	4	13		
		Pustules	0	4	3	1	8		
19	R	Comedones	12	10	8	10	40	90	4
		Microcysts	3	10	15	4	32		
	I	Papules	1	5	3	3	12		
		Pustules	2	3	0	1	6		
20	R	Comedones	0	4	4	3	11	30	4
		Microcysts	6	3	1	0	10		
	I	Papules	2	2	4	1	9		
		Pustules	0	0	0	0	0		
21	R	Comedones	2	4	3	3	12	55	4
		Microcysts	5	11	12	6	34		
	I	Papules	0	3	2	2	7		
		Pustules	0	1	1	0	2		
22	R	Comedones	4	5	6	9	24	60	4
		Microcysts	7	5	7	2	21		
	I	Papules	3	3	4	0	10		
		Pustules	1	2	2	0	5		
23	R	Comedones	7	5	10	5	27	67	4
		Microcysts	13	8	6	3	30		
	I	Papules	2	1	2	1	6		
		Pustules	1	1	2	0	4		
24	R	Comedones	9	4	3	4	20	62	4
		Microcysts	17	4	6	3	30		
	I	Papules	1	4	1	1	7		
		Pustules	0	1	3	1	5		
25	R	Comedones	4	2	2	4	12	34	4
		Microcysts	2	1	1	3	7		
	I	Papules	2	2	3	2	9		
		Pustules	0	3	2	1	6		
26	R	Comedones	6	6	5	3	20	52	4
		Microcysts	7	4	3	6	20		
	I	Papules	0	5	5	0	10		
		Pustules	1	1	0	0	2		
27	R	Comedones	5	9	3	5	22	92	5
		Microcysts	5	12	12	5	34		
	I	Papules	1	9	7	4	21		
		Pustules	0	8	4	3	15		
28	R	Comedones	20	14	13	2	49	157	6
		Microcysts	20	3	11	4	38		
	I	Papules	3	16	20	14	53		

		Pustules	2	7	4	4	17		
29	R	Comedones	4	15	7	5	31	81	5
		Microcysts	5	10	5	7	27		
	I	Papules	2	6	4	2	14		
		Pustules	1	4	2	2	9		

[1] R=Retentional acne lesions; [2] I=Inflammatory acne lesions

* EXG score=0: No acne at all – No comedones or other signs of acne
EXG score=1: Comedonal acne – Open comedones (non-irritated)
EXG score=2: Mild inflammatory acne – Open and closed comedones and papules (mild)
EXG score=3: Mild to moderate inflammatory acne – Open and closed comedones, papules and pustules (only a few, mild)EXG score=4: Moderate inflammatory acne – Open and closed comedones, papules and pustules (several, moderate)
EXG score=5: Moderate nodulocystic acne – Open and closed comedones, papules and pustules (many, severe)
EXG score=6: Severe nodulocystic acne – Open and closed comedones, papules and pustules (many, severe, scars)
